# Genetical genomic determinants of alcohol consumption in rats and humans

**DOI:** 10.1186/1741-7007-7-70

**Published:** 2009-10-27

**Authors:** Boris Tabakoff, Laura Saba, Morton Printz, Pam Flodman, Colin Hodgkinson, David Goldman, George Koob, Heather N Richardson, Katerina Kechris, Richard L Bell, Norbert Hübner, Matthias Heinig, Michal Pravenec, Jonathan Mangion, Lucie Legault, Maurice Dongier, Katherine M Conigrave, John B Whitfield, John Saunders, Bridget Grant, Paula L Hoffman, WHO/ISBRA Study on State and Trait Markers of Alcoholism

**Affiliations:** 1 Department of Pharmacology, University of Colorado, Denver, Aurora, CO, USA; 2 Department of Pharmacology, University of California San Diego, La Jolla, CA, USA; 3 Department of Pediatrics, University of California Irvine, Irvine, CA, USA; 4 Laboratory of Neurogenetics, National Institute on Alcohol Abuse and Alcoholism, Bethesda, MD, USA; 5 Committee on the Neurobiology of Addictive Disorders, The Scripps Research Institute, La Jolla, CA, USA; 6 Department of Biostatistics and Informatics, Colorado School of Public Health, University of Colorado Denver, Aurora, CO, USA; 7 Institute of Psychiatric Research, Indiana University School of Medicine, Indianapolis, IN, USA; 8 Max Delbrück Center for Molecular Medicine, Berlin, Germany; 9 Institute of Physiology, Czech Academy of Sciences, Prague, Czech Republic; 10 MRC Clinical Sciences Centre, London, UK; 11 Department of Psychiatry, McGill University, Montreal, Quebec, Canada; 12 Drug Health Services, Royal Prince Alfred Hospital, Sydney Medical School, University of Sydney, New South Wales, Australia; 13 Queensland Institute of Medical Research, Queensland, Australia; 14 School of Medicine, University of Queensland, Brisbane, Queensland, Australia; 15 Division of Epidemiology, National Institute on Alcohol Abuse and Alcoholism, Rockville, MD, USA; 16 Department Psychology-Neuroscience, University of Massachusetts Amherst, Amherst, MA, USA; 17 Applied Biosystems, Lingley House, 120 Birchwood Blvd., Warrington, Cheshire, WA3 7QH, UK

## Abstract

**Background:**

We have used a genetical genomic approach, in conjunction with phenotypic analysis of alcohol consumption, to identify candidate genes that predispose to varying levels of alcohol intake by HXB/BXH recombinant inbred rat strains. In addition, in two populations of humans, we assessed genetic polymorphisms associated with alcohol consumption using a custom genotyping array for 1,350 single nucleotide polymorphisms (SNPs). Our goal was to ascertain whether our approach, which relies on statistical and informatics techniques, and non-human animal models of alcohol drinking behavior, could inform interpretation of genetic association studies with human populations.

**Results:**

In the HXB/BXH recombinant inbred (RI) rats, correlation analysis of brain gene expression levels with alcohol consumption in a two-bottle choice paradigm, and filtering based on behavioral and gene expression quantitative trait locus (QTL) analyses, generated a list of candidate genes. A literature-based, functional analysis of the interactions of the products of these candidate genes defined pathways linked to presynaptic GABA release, activation of dopamine neurons, and postsynaptic GABA receptor trafficking, in brain regions including the hypothalamus, ventral tegmentum and amygdala. The analysis also implicated energy metabolism and caloric intake control as potential influences on alcohol consumption by the recombinant inbred rats. In the human populations, polymorphisms in genes associated with GABA synthesis and GABA receptors, as well as genes related to dopaminergic transmission, were associated with alcohol consumption.

**Conclusion:**

Our results emphasize the importance of the signaling pathways identified using the non-human animal models, rather than single gene products, in identifying factors responsible for complex traits such as alcohol consumption. The results suggest cross-species similarities in pathways that influence predisposition to consume alcohol by rats and humans. The importance of a well-defined phenotype is also illustrated. Our results also suggest that different genetic factors predispose alcohol dependence versus the phenotype of alcohol consumption.

## Background

The term "genetical genomics" is entering the common parlance of researchers, denoting the combined use of genetic marker information and transcriptome analysis. Complex trait phenotyping can be fruitfully combined with genetical genomic analysis to ascertain the candidate genes and gene product interaction pathways which significantly influence the variation in expression of a phenotype of interest. We and others have utilized the genetical genomics and phenomic approaches to identify genes and pathways important in genetically influenced complex traits such as obesity, respiratory function and "addictive" behavior [1-3]. In the area of addictive behavior, our laboratory has focused on certain endophenotypes, including the genetic contributors to acute functional tolerance to ethanol [4,5] and alcohol preference in mice [2]. These studies produced results that implicated the protein products of genes important in learning and memory as contributors to acute functional tolerance to ethanol, and proteins important in orosensory systems and information processing as being important in alcohol preference in mice.

The genus/species *Rattus norvegicus *has been used extensively in studies of addictive behavior, not only with regard to ethanol, but also to cocaine, opiates, cannabinoids, nicotine, etc. [6,7]. Although several research groups have suggested that similar or identical biochemical systems in particular brain areas mediate self-administration of addictive drugs [6,8-10], such proposals have not been tested by applying the unbiased approach of genetical genomics, combined with phenotyping, to studies with rats. In the present study, we have used the HXB/BXH recombinant inbred rat strains [11], which represent a unique resource for alcohol research. These rats have previously been used for quantitative trait locus (QTL) analysis of various cardiovascular phenotypes and metabolic and behavioral traits [11]. We have generated brain transcriptome data for 28 recombinant inbred strains of HXB/BXH rats [11], and have combined these data with genetic marker data for these animals [12]. We have also tested the rats in a standard procedure for measuring alcohol preference [13] and these data have allowed us to generate insight into a complex of genes and their protein products which have significant association with the trait of alcohol preference/consumption in rats.

We have also taken the opportunity to directly compare the candidate genes and pathways for alcohol consumption that we identified using the rodent model, to candidate genes in human populations. We used the "Addictions Array" [14] to assess the relationships among a panel of genetic markers (SNPs) for "alcoholism and addiction" candidate genes, and the phenotype of alcohol consumption, in two populations of humans that had been characterized in the WHO/ISBRA Study on State and Trait Markers of Alcohol Dependence [15]. The results of this analysis demonstrate a convergence of the human and animal results and provide a differentiation between the genetic polymorphisms associated with predisposition to alcohol drinking and the susceptibility to alcohol dependence.

## Methods

### Animal Studies

#### Animals

Male rats from well-characterized HXB/BXH recombinant inbred (RI) rat strains [11] were used for these studies. The rats were rederived into and maintained in a colony at the University of California, San Diego. These rats were developed from an intercross between two inbred strains, the Wistar origin spontaneously hypertensive rat (SHR/Ola) and Brown Norway congenic (BN-Lx/Cub) [11]. The RI strains replicate the F2 generation of the intercross and have been brother-sister mated for more than 90 generations. Development of the RI strain set utilized gender reciprocal crossing, providing two strain sets that differ in the source of mitochondrial DNA and the Y chromosome. The HXB rats carry mitochondrial DNA from the SHR rats and the Y chromosome from the BN-Lx rats, while the BXH strains are the reverse.

#### Alcohol Consumption

Data on alcohol consumption were gathered on 23 HXB/BXH strains and the two progenitor strains. The number of rats per strain ranged from 9 to 12, with 242 total rats being utilized to measure alcohol consumption. In the first week (week 0) of treatment, rats were given 10% ethanol as their only choice of fluid. For the next seven weeks (week 1 - week 7), the rats were given a choice of two bottles, one with water and one with a 10% (v/v) ethanol solution [13]. Volumes of water and alcohol solution consumed were measured by weight on Monday, Wednesday and Friday of each week. The placement of the two bottles was varied after each measurement to avoid a place preference effect. For the current study we used alcohol consumption data from the second week of the two-bottle choice paradigm. These data were chosen to reflect a stable level of alcohol consumption/"preference" and to facilitate comparisons with alcohol consumption/preference in rats selectively bred for this trait [7], which use a similar paradigm for selection. The stability of this measure was confirmed by comparison of the mean levels of alcohol consumption for each strain during week 2 and week 7 of the two-bottle choice procedure. A correlation analysis resulted in an r^2 ^= 0.62 (*P *< 0.001).

#### Behavioral QTL Analysis

Behavioral QTLs (bQTLs) were calculated for the alcohol consumption phenotype using strain means for average daily alcohol consumption in grams per kilogram during week 2 of the two-bottle choice period. Individual values were not included if they were more than two standard deviations from the strain mean. The recently developed STAR Consortium SNP set of genetic markers was used in this QTL analysis [12]. There were 21 recombinant inbred strains plus the two progenitor strains that had both alcohol consumption data and SNP data. Using these 23 strains, 962 unique strain distribution patterns were identified in the STAR Consortium SNP set. Strain means for alcohol consumption were used in a marker regression QTL analysis. QTLs with empirical *P*-values less than 0.05 [based on permutation, [16]] were considered significant, and markers with logarithm of odds (LOD) scores above 2.0 were considered "suggestive" and were included as potential bQTL for alcohol consumption. The 20 Mb region around the significant and suggestive markers was used as the bQTL interval. bQTL analyses were conducted using the R/qtl package in R [17].

#### Microarray Analysis

Naive (non alcohol-exposed) male HXB/BXH RI rats were used for microarray analysis. Rats were group-housed and individual rats were quickly anesthetized with isofluorane/air and decapitated according to a protocol approved by the UCSD IACUC. Brains were rapidly removed, sectioned sagittally into two hemispheres, and frozen on dry ice or in liquid nitrogen. The right half-hemispheres were promptly shipped on dry ice to the University of Colorado, where they were kept at -80°C until used for RNA extraction. CodeLink Rat Whole Genome Bioarrays were obtained from G.E. Healthcare/Amersham Biosciences (Piscataway, NJ).

##### Total RNA Extraction

RNA from the right halves of brains of five to seven rats per strain (26 RI strains and the progenitor strains) was used for these experiments (our prior studies and those of others [18] showed no statistically significant differences in gene expression between left and right halves of rodent brain). Brains were homogenized in lysis buffer using a Polytron (Brinkmann Instruments, Westbury, NY, USA), and total RNA was isolated using the RNeasy Midi Kit (Qiagen) (Valencia, CA, USA) following the protocol supplied by the manufacturer. An additional clean-up of total RNA was carried out using the RNeasy Mini Kit (Qiagen). Quality of the samples was assessed on an Agilent Bioanalyzer (Agilent Technologies, Santa Clara, CA, USA).

###### Expression Analysis Using CodeLink Rat Bioarrays

Each CodeLink Rat Whole Genome Bioarray used in these experiments has ~34,000 probes for rat transcripts and expressed sequence tags (ESTs), plus a number of positive and negative control probes. Using the protocol supplied by the manufacturer, double-stranded cDNA was synthesized from the total RNA (4 μg of total RNA from an individual rat brain was used for each array) and was used to obtain biotin-labeled cRNA by an *in vitro *transcription reaction. Biotin-labeled cRNA was recovered using the RNeasy kit (Qiagen) and concentration and purity were assessed spectrophotometrically. Biotin-labeled cRNA from each rat was then fragmented and hybridized with an individual CodeLink Bioarray. The Bioarrays were subsequently stained for 30 min with Cy5-streptavidin and washed before scanning. Arrays were scanned using a GenePix 4000B Scanner [Axon Instruments (Molecular Devices Corp), Union City, CA, USA] and the images were quantitated with CodeLink Expression Analysis (v 4.1) software (Amersham Biosciences).

####### Quality Control and Normalization

Raw intensity values were obtained from the CodeLink processing software. Entire arrays were examined for quality control purposes both before and after normalization. Before normalization, arrays were examined by boxplots and coefficient of variation (CV) plots for consistency, along with background levels and proportion of probes within each category for the CodeLink quality flags. After normalization, samples were examined using hierarchical clustering and pair-wise scatter plots to identify samples that severely deviated from other samples within the same strain (such samples were eliminated from further analysis).

In preparation for normalization, probes were removed from the datasets if they were one of the negative or positive controls placed on the array by the manufacturer. Next, individual values were eliminated based on the quality flags assigned by the CodeLink Expression Analysis Software. Values were eliminated if they were flagged as M (spot was identified to be defective through image inspection at manufacturing), C (spot has a high level of background contamination), I (spot has an irregular shape), or S (spot has a high number of saturated pixels). Values were retained if they were flagged G (spot is good) or L (spot is below local background noise). In addition, to be able to take the log base 2 transformation of the background adjusted intensity values, all background adjusted intensity values below zero were replaced with the value 0.00001. The data were then normalized using a cyclic LOESS (locally weighted scatterplot smoothing) procedure executed in R which accounted for the missing intensity values. Normalization and quality control were executed in R using the affy, codelink, and limma packages [19].

#### Identification of Candidate Genes for Alcohol Consumption

After normalization of the expression data, several filters were applied in order to identify candidate genes for alcohol preference in the HXB/BXH RI strains.

##### Annotation Filter

Probes were eliminated from further analysis if they did not represent a valid gene. Each probe is represented by a 30-mer base sequence. These 30-mers were compared to the most recent (November 2004) assembly of the rat genome using the BLAST-like alignment tool (BLAT) available from the UCSC website [20]. A probe was retained if at least 28 of the bases matched a region in the genome with less than a two base gap within the sequence. This region also had to fall within an established exon for a RefSeq rat gene, or within an exon sequence for a RefSeq mouse or human gene whose entire sequence matched a region in the rat genome.

##### Expression QTL analysis

Expression QTL (eQTL) were established using the HXB/BXH RI brain gene expression data. For this analysis, 26 strains (2 progenitor strains and 24 RI strains) were represented among 124 samples (data from two strains for which genotyping was not available were not included). The quality control measures and normalization procedures described above were utilized. The STAR marker set described in the behavioral QTL methods section was also utilized in this analysis. Because more strains were included in this analysis than in the bQTL analysis, 1,184 unique strain distribution patterns were represented in this marker set.

To calculate eQTLs, mean expression levels within strains were used as phenotypic values in a QTL analysis implemented in QTLReaper, which is written in C and compiled as a Python module. A weighted marker regression analysis was used within this program to calculate likelihood ratio statistic (LRS) scores for each marker. LRS scores were transformed to LOD scores for convenience by dividing by 4.61. The regression is weighted to account for the different number of arrays within strains used to calculate strain means. The weight is based on the repeatability of the transcript intensity and number of arrays used to calculate the strain mean [21]. The empirical *P*-value with respect to the maximum LOD score was calculated for each transcript by permutation [16]. The number of permutations per transcript was increased until the maximum LOD score from the true data was no longer in the top ten of LOD scores from the permutation, or until 1,000,000 permutations were calculated. For eQTLs with empirical *P*-values less than 0.10, 95% confidence limits for location were calculated using 1,000 bootstrap samples [22]. Probes were retained if they had a significant (*P *< 0.05) or suggestive (*P *< 0.10) eQTL.

###### eQTL/bQTL overlap filter

Probes whose eQTL 95% confidence interval for genomic location overlapped the chromosomal location of any of the four behavioral QTLs were retained for further consideration.

###### Heritability Filter

A broad-sense heritability was calculated for each probe in the expression data used in the correlation analysis. Probes were retained if they had a broad sense heritability ≥ 50%.

###### Detection Limit Filter

Detection limits are calculated by the CodeLink software as the median intensity of the local background plus 1.5 local background standard deviations. Probes were eliminated if values were below detection limits for ≥ 50% of the samples.

###### Correlation Filter

The correlation of alcohol consumption and gene expression was modeled using a joint mixed model for both the expression and alcohol consumption data, *Y*_*ijk *_= *μ*_*k *_+ *s*_*ik *_+ *ε*_*ijk *_for *i *= 1,...,25 strains, *j *= 1,...n_ik _rats, and *k *= 1,2 where k = 1 indicates a gene expression measure and k = 2 indicates an alcohol consumption measure. The ε_ijk _are independent and identically distributed from a normal distribution with a 0 mean and a measure-specific variance (σ_k_^2^). The s_ik _are random strain-measurement effects. The pair, [s_i1_, s_i2_], are independent and identically distributed as a bivariate normal with an unstructured covariance where a separate between-strain variance is calculated for expression and alcohol consumption plus a covariance between expression and alcohol consumption. This model was executed in SAS version 9.1 using a maximum likelihood model in the linear mixed model function called PROC MIXED. A likelihood ratio statistic was used to determine if the covariance between gene expression and alcohol consumption is significantly different from zero. Probes which demonstrated an overlap of their eQTL location and the genomic location of one of the four bQTLs and which passed the other filters listed above, were used in our correlation analysis. Probes were retained if they had a significant covariance with alcohol consumption (*P *< 0.05).

### Proportion of Variance Explained and Model Building

Once a list of candidate genes was established, the proportion of genetic variance in alcohol consumption among strains that was explained by gene expression was examined. A multivariate genetic model for alcohol consumption was constructed using a linear regression with strain means for both gene expression and alcohol consumption. Genes were selected for inclusion using a forward stepwise selection process with a significance criterion of 0.01 for entrance into the model. A forward selection method was chosen over a backward selection method in this instance because of the large number of potential covariates (candidate genes). The proportion of genetic variance explained was determined for each candidate gene individually and for the combination of transcripts in the final multivariate genetic model which described the strain variation in alcohol consumption.

### Functional Analysis of Candidate Gene Products

In order to determine the functions of the candidate gene products, and assemble them into pathways, a literature search was performed using PubMed. The gene symbols (NCBI) and gene product names were used as key words.

### Replication Studies

In order to replicate differences in gene expression associated with alcohol consumption, gene expression profiles were analyzed using Affymetrix GeneChip Rat Gene 1.0 ST arrays (Affymetrix, Santa Clara, CA, USA), which utilize probesets that span the coding region of each gene. RNA from brains of five male rats from HXB RI strain 23 (low alcohol consumption) and five male rats from HXB RI strain 26 (high alcohol consumption) was used for these studies. These particular strains were chosen because the expression levels of all of the candidate genes (assayed on the CodeLink arrays (Amersham Biosciences)) showed an appropriate direction of differential expression, compared to the correlation analysis, although not all of the differences in the candidate gene expression were statistically significant between these particular strains. RNA (0.3 μg per rat) was processed and hybridized to the Affymetrix arrays (cDNA from one rat to each individual array) according to the manufacturer's protocol. Arrays were labeled and scanned as previously described [2]. For data analysis, raw data and the normalized probeset data were assessed for quality using the 'aroma.affymetrix' package in R [23] and Expression Console from Affymetrix [24]. All ten arrays passed quality control. Raw intensity values for individual probes were corrected for background using the RMA correction, probe values were quantile normalized, and probes were summarized into probesets [25] in aroma.affymetrix. The probe intensities were compared between the two strains using a two-sample t-test assuming unequal variance on both the probeset and the probe level. Probe level data were only examined for those probes that fell within the same exon, upstream, or downstream region as the original CodeLink probe.

### Human Association Study

#### Population

Subjects for the human genetics association study were recruited as part of the WHO/ISBRA Study on State and Trait Markers of Alcohol Use and Dependence [15]. More specifically, phenotypic data from Caucasian subjects from Montreal, Canada and Sydney, Australia were analyzed since these subjects were also genotyped using the Addiction Array [14]. The phenotypic data were collected using a structured interview instrument that was developed for the WHO/ISBRA Study. This instrument was developed in concert with the development of the Alcohol Use Disorders and Associated Disabilities Interview Schedule (AUDADIS), and included eight major sections [see [15]]. Of particular relevance for the current study, the interview included a section that gathered information on beverage-specific frequency and quantity of alcohol drinking during the past 30 days, and the symptoms experienced with various levels of drinking. Another section gathered information that allowed for International Classification of Diseases -10 (ICD-10) and Diagnostic and Statistical Manual -IV (DSM-IV) diagnosis of alcohol abuse, dependence and withdrawal syndromes. In addition, family history of alcohol problems or alcohol dependence, major depression, drug use and abuse and other psychiatric problems were assessed (see Additional file 1, Table S2). Additional information on the demographic characteristics of the human subjects is contained in Additional file 1, Table S3. For more details on the original study and data collection, see Glanz et al. [15].

#### SNPs

Genotype data were obtained from a custom array using the Illumina Goldengate SNP technology assay platform. This array includes a panel of markers designed to extract full haplotype information for 130 candidate genes associated with alcoholism, other drug addictions, and mood and anxiety disorders. The design and performance of this array are described in detail by Hodgkinson et al. [14]. Briefly, the array contains 1,350 tagSNPs representing the 130 addiction-related genes, and an additional 186 markers identified as highly informative for ancestry (AIMs).

#### Outcome Measure

The main phenotypic outcome measure of this analysis was alcohol consumption reported in grams per kilogram of body weight per day. The total amount of alcohol consumed in the last 30 days was calculated based on information that was collected in the WHO/ISBRA questionnaire on beverage-specific frequency and quantity of drinking and this total was divided by 30 to obtain the daily average.

#### Quality Control

Prior to the univariate analyses, the SNP dataset was subjected to strict quality control standards. Individual SNPs were eliminated if they were not in Hardy-Weinberg equilibrium (FDR<0.01) or if they had minor allele frequency less than 5%. Subjects were eliminated if their genotype was not determined for at least 80% of the SNPs according to standards set forth in Hodgkinson et al. [14]. The ancestry informative markers were not considered in any of the association analyses with the exception of the tests for stratification.

#### Population Stratification

Since the subjects came from two distinct geographical locations, it is reasonable to consider that there may be some genetic differences between these populations. Population stratification with respect to the outcome, alcohol consumption, was tested against the AIMs using the concept outlined in Pritchard and Rosenberg [26]. A Fisher's combined probability test was used to calculate significance, due to the continuous nature of the outcome. Differences in allele frequencies between the two populations for the AIMs were also tested using Pritchard and Rosenberg's [26] method of combining chi-square statistics.

#### Univariate Analyses

The association between alcohol consumption and each individual SNP was tested in an ANOVA using a genotype model. *P-*values were adjusted using a false discovery rate (FDR). SNPs with an FDR < 0.05 were considered to show a significant association.

#### Haplotypes

Haplotype blocks were determined in Haploview [27] for genes with significant SNPs, using the Gabriel et al. [28] method for identifying block boundaries. Haplotype block structure was examined in the WHO/ISBRA population that had the significant SNP association, and in the CEPH (Utah Residents with Northern and Western European Ancestry) population in the HapMap project [29] for comparison. The haplotype block structure in the HapMap population was used to construct haplotypes for individual subjects. Subjects were assigned haplotype pairs and the posterior probabilities of those haplotype pairs were determined using haplo.em in the haplo.stats package of R. If haplotypes had less than five counts, they were recoded to the closest haplotype. If the closest haplotype differed by more than one SNP, data related to the minor haplotype were deleted. All further analyses were weighted by the posterior probability of that particular haplotype pair. Additive, recessive, and dominant representations for all haplotypes were examined in a forward selection model building procedure where the criterion for entrance into the model was *P *< 0.01.

#### Multivariate Genetic Model

A backwards selection linear regression was used to create a multivariate model that potentially could include effects for all genes that were significant in the univariate analysis. To start the model selection procedure, the best fit univariate model for each gene, either haplotype-based or SNP-based, according to the Bayesian Information Criterion [BIC, 30], was entered into the model. An exit criterion of *P *> 0.01 was used in the model building process.

#### Multivariate Model With Covariates

A multivariate model was also created using the candidate genes and the covariates shown in Additional file 1, Table S2. A backwards selection model building process was used, with a more conservative exit criterion of *P *> 0.001.

#### Association Between Covariates in Final Multivariate Model and Candidate Genes

Each candidate gene was tested for association with each of the covariates that remained in the final multivariate model. Each gene by covariate pair was tested independently. Genes were represented using their best fit univariate model as described above. For the binary covariates, a Fisher's exact test was used for association. For continuous covariates, an ANOVA model was used for association. All *P*-values were adjusted (within population) for multiple comparisons using an FDR [31].

## Results

### Animal Studies

#### Alcohol Consumption by HXB/BXH RI Rat Strains

**Figure 1:**
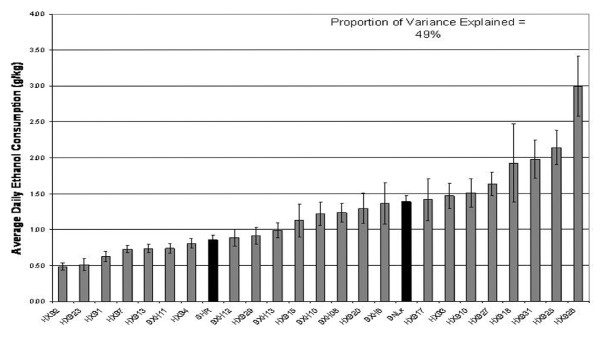
**Strain Distribution of Average Daily Ethanol Consumption**. Rats were given 10% ethanol as their only choice of fluid for one week (week 0). For the next seven weeks, rats were given a choice of two bottles, one with water and one with a 10% ethanol solution. The data shown are mean ± SEM of average daily ethanol consumption (g/kg body weight) during the second week of the two-bottle choice paradigm.

Figure 1 shows the average daily alcohol consumption for each of the HXB/BXH RI strains and the two progenitor strains. Alcohol consumption varied among strains, and it was calculated that 49% of the variance could be attributed to strain (genetic variance).

##### Behavioral QTL

**Figure 2:**
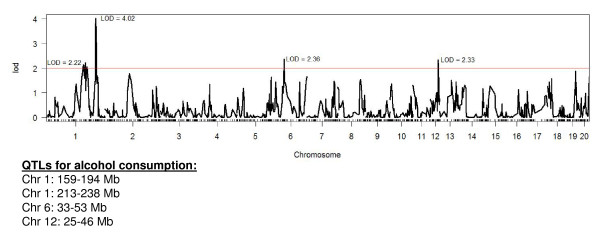
**QTLs for Alcohol Consumption by HXB/BXH RI Rat Strains**. Behavioral QTLs (bQTLs) were calculated using the data shown in Figure 1. Individual values were not included if they were more than two standard deviations from the strain mean. The STAR Consortium SNP markers (The STAR Consortium, 2008) were used for this analysis. There were 21 RI strains and the two progenitor strains that had both alcohol consumption and SNP data. Strain means for alcohol consumption were used in a marker regression QTL analysis, which was conducted using the R/qtl package in R. The 20 Mb region around the suggestive markers was used as the bQTL interval shown.

Four genomic regions were identified as having significant or suggestive associations with alcohol consumption in the HXB/BXH rats (Figure 2). The most significant marker was on chromosome 1 at 224.7 Mb, and it had a LOD score of 4.02 (empirical *P*-value = 0.05). This particular QTL and two other regions had multiple adjacent markers with a LOD score above 2. The bQTL region in each case was extended 10 Mb on either side of the adjacent markers which had LOD scores above 2.0 (Figure 2).

### Gene Expression Data and Filtering of Gene Expression Data from HXB/BXH RI Strains

#### Quality Control and Normalization

After quality control , 121 arrays remained from the 25 strains used for the alcohol consumption and gene expression correlation analysis. We eliminated 497 control probes from further analysis along with a total of 31,379 (<1%) individual expression values due to quality control flags. In addition 45,722 expression values (1% of remaining values) were changed to 0.00001 because original (local background-corrected) spectroscopically derived values were less than 0. A total of 33,849 probes on each of the 121 arrays were included in the further analysis (filtering and correlation analysis).

#### Filtering Procedures

As noted in methods, a number of filters were used to identify a list of potential candidate genes for alcohol preference in the HXB/BXH RI rats. Starting with the quality controlled, normalized dataset, we initially determined probes that could be matched with exonic regions of the rat genome (or with a homologous human or mouse sequence). There are 14,622 unique rat RefSeq DNA IDs specified by the UCSC Genome Browser. Of these, 8,433 genes (10,162 probes) are included on the CodeLink Whole Genome Rat Array. We identified 4,966 additional probes on the CodeLink array that were associated with either a human or mouse homolog gene (RefSeq DNA ID) whose sequence matched an area of the rat genome. Of these 15,128 probes, 12,466 (82%) were present (by our criteria) in the brain samples that we analyzed. Significant or suggestive eQTLs could be identified for 2,164 probes (17%), and, of these, 378 probes had an eQTL that overlapped one of the bQTLs for alcohol consumption in these rats. Transcripts with eQTLs that overlap bQTLs were considered to represent likely candidate genes for the alcohol consumption behavior [2]. The heritability filter was used to insure that the expression levels of genes in the candidate gene list had a high heritability, and 308 probes met the criterion of a broad sense heritability ≥ 0.5. We also applied a filter based on detectable levels of expression in the rat brains. Only transcripts that were expressed in brains of ≥ 50% of the individual samples were included. Although this choice is arbitrary, we did not wish to include genes with low or undetectable levels of brain expression in a large number of strains, as such transcripts could produce unreliable results. This filter was passed by 304 probes. The final filter was correlation of probe expression with alcohol consumption. By initially applying all of the previous filters, the risk of false positives in this correlation step was reduced. Of the 304 starting probes, 20 had a significant (*P *< 0.05) correlation with alcohol consumption in the HXB/BXH RI rat strains. These probes are listed in Table 1.

**Table 1:** Candidate Genes for Alcohol Consumption by HXB/BXH RI Rats.

**Gene Symbol**	**Gene Description**	**Gene Location Chr(Mb)**	**eQTL Location Chr(Mb)**	**eQTL Mb Range**	**eQTL LOD (p-value)**	**bQTL Location Chr(Mb)**	**Correlation Coefficient (p-value)**	**Broad Sense Heritability**	**Percent Present**
Cckbr	cholecystokinin B receptor	1 (163.17)	1 (168.95)	139.73-179.97	6.3 (<0.001)	1 (159-194)	0.61 (0.0023)	0.82	100%
		
Coq7	demethyl-Q 7 (Coenzyme q (ubiquinone) biosynthetic enzyme (DHPB methyltransferase) 7)	1 (176.76)	1 (177.64)	152.25-179.75	9.0 (<0.001)		0.55 (0.0059)	0.93	100%
		
Fgfr2	fibroblast growth factor receptor 2 isoform c	1 (189.48)	1 (184.63)	173.38-184.63	20.7 (<0.001)		0.58 (0.0029)	0.99	93%
		
Ptpre	protein tyrosine phosphatase receptor type E	1 (195.16)	1 (195.13)	184.63-196.57	13.1 (<0.001)		-0.43 (0.0348)	0.95	100%
		
Abat	4-aminobutyrate aminotransferase	10 (7.04)	1 (132.04)	12.84-249.11	4.2 (0.027)		-0.51 (0.0267)	0.56	100%
		
Tpst1	tyrosylprotein sulfotransferase 1 (predicted)	12 (27.59)	1 (15.55)	9.02-205.67	3.1 (0.096)		0.51 (0.0265)	0.54	99%

Tmem2	transmembrane protein 2	1 (225.04)	1 (223.19)	39.64-254.91	3.7 (0.065)	1 (213-238)	0.53 (0.0095)	0.85	100%

Mboat2	membrane bound O-acyltransferase domain	6 (42.49)	6 (38.84)	18.53-131.90	3.5 (0.072)	6 (33-53)	0.43 (0.0455)	0.73	100%
		
Dynlrb1	dynein light chain roadblock-type 1	3 (145.72)	6 (38.84)	18.53-115.00	3.6 (0.095)		0.44 (0.0456)	0.66	100%
		
Tnk2	tyrosine kinase, no-receptor, 2	11 (69.93)	6 (25.68)	0.27-38.84	4.0 (0.010)		-0.50 (0.0212)	0.68	100%
		
Nek3	NIMA (never in mitosis gene a)-related expressed	16 (74.52)	6 (37.98)	25.56-37.98	4.1 (0.008)		-0.43 (0.0499)	0.69	97%
		
Afap1l1	actin filament associated protein 1-like 1	18 (57.71)	6 (33.80)	25.68-50.93	3.3 (0.090)		0.49 (0.0227)	0.74	100%
		
Mc4r	melanocortin 4 receptor	18 (63.39)	6 (33.80)	33.80-50.93	3.7 (0.006)		0.46 (0.0417)	0.59	96%
		
Tubb6	tubulin beta 6	18 (63.92)	6 (33.80)	33.80-124.56	3.2 (0.097)		0.47 (0.0344)	0.63	98%
		
Spire1	spire homolog 1	18 (64.02)	6 (33.80)	18.53-98.70	3.4 (0.025)		0.53 (0.0109)	0.77	100%

Mogat2	monoacyglycerol O-acyltransferase 2	1 (156.52)	12 (34.31)	29.92-38.52	3.5 (0.028)	12 (25-46)	-0.45 (0.0353)	0.78	98%
		
F2rl1	proteinase-activated receptor-2 G protein coupled	2 (25.89)	12 (38.52)	36.18-43.30	2.8 (0.059)		0.53 (0.0224)	0.52	52%
		
P2rx4	P2X purinoceptor 4 (ATP receptor)	12 (34.94)	12 (34.50)	13.40-34.50	5.1 (0.003)		-0.70 (0.0004)	0.80	100%
		
Rab35	RAB35 member RAS oncogene family	12 (42.23)	12 (41.98)	38.21-43.30	9.0 (<0.001)		0.45 (0.0296)	0.91	100%
		
Pop5	processing of precursor 5 ribonuclease P/MRP	12 (42.64)	12 (41.98)	36.18-43.30	4.4 (0.025)		-0.54 (0.0125)	0.68	100%

All transcripts listed displayed an eQTL/bQTL overlap, showed a significant correlation with alcohol consumption, and passed the filters for heritability and detectability of expression.

The chromosomal locations, as well as the location of the bQTLs and eQTLs for the 20 probes, are shown in Table 1. Nine of the candidate genes were posited to be *cis*-regulated, i.e., the chromosomal location of the gene is within 5 Mb of the eQTL location. The expression of the rest of the candidate genes was posited to be regulated from an area in the genome distant from the physical location of the gene (*trans*-regulation). The expression of seven of the *trans*-regulated genes was controlled from an eQTL on chromosome 6. This finding suggests the possibility that there is, for example, a polymorphic gene coding for a transcription factor within this eQTL region, and that this transcription factor contributes to the regulation of the expression of all seven of the candidate genes. Although 18 putative transcription factor genes could be identified in the eQTL region on chromosome 6 (see Additional file 1, Table S1), there is not sufficient information available on the exact nature of the binding sites for these transcription factors to determine whether such binding sites are overrepresented in the promoter regions of the *trans*-regulated genes. Table 1 also shows the correlation coefficients for the correlation with alcohol consumption, and the results of the heritability and expression level filters. The expression levels of 13 of these probes were positively correlated with alcohol consumption, and expression levels of seven were negatively correlated.

The transcript that explains the greatest proportion of genetic variance in alcohol consumption in the HXB/BXH RI strains is *P2rx4 *(r^2 ^= 0.39, model *P *= .0009). When we performed a forward stepwise regression analysis to determine a multivariate model for alcohol consumption, the final model contained *P2rx4 *and *Tmem2*. The model explained 57% of the genetic variance among strain means for alcohol consumption.

#### Replication Study of Candidate Gene Expression in HXB/BXH Rat Strains

The results obtained using the CodeLink arrays were assessed using a different gene expression measurement platform. The analysis of the Affymetrix Array data for the expression levels of the candidate genes in brains of rats from two HXB/BXH strains are shown in Table 2. These two strains were chosen based on their differences in voluntary alcohol consumption (see Figure 1) and their differences in brain candidate gene expression levels determined using the CodeLink arrays. Table 2 includes data from the CodeLink and Affymetrix arrays for these two strains to allow comparisons. In evaluating the replicability of results, it is necessary to keep in mind that even though the candidate genes displayed a significant correlation with alcohol consumption when all RI strains were considered, not all of the candidate genes (based on CodeLink results) displayed significantly different expression levels in these two particular strains. However, the expression differences in these strains were all in the same direction as the overall correlation. Therefore, in comparing to the results for the Affymetrix arrays, we took into account both the significance and direction of expression differences. Three candidate genes displayed significant differences in expression, in the same direction, using both arrays: *Afap1l1*, *Cckbr *and *P2rx4*. Another six candidate genes showed significant differences using CodeLink arrays, and the same direction of differences on the Affymetrix arrays (*Tnk2*, *Tubb6*, *Ptpre*, *Mogat2*, *Nek3 *and *Tmem2*). Three of the candidate genes did not show significant differences in expression using the CodeLink arrays, but either did show a significant difference in expression levels in the same direction (*Mc4r*), or showed non-significant differences in expression in the same direction using the Affymetrix arrays (*F2rl1*, *Abat*). Based on this analysis, we placed more confidence in these 12 of the 20 original candidate genes. The rest of the candidate genes did not show significantly different levels of expression using the Affymetrix arrays, and showed differences in expression in opposite directions on the two platforms. Since the expression values obtained with CodeLink arrays are based on one probe, while the expression values from the Affymetrix arrays represent a summary from multiple probes, spread throughout each gene, we also compared results for probes on the Affymetrix arrays that correspond in location to the probes on the CodeLink arrays. In most cases, the results for Affymetrix individual probes and probesets were the same. For *Tnk2*, the result from the individual probe on the Affymetrix array indicated a significantly different expression level between strains in the same direction as had been determined using the CodeLink array between the tested strains. We have limited our discussion of candidate genes to those that were found (same direction of differential expression) using both CodeLink and Affymetrix arrays (Table 2).

**Table 2:** Replication Study of Candidate Genes

	**Affymetrix Results**				**CodeLink Results**				
**Gene Symbol**	**HXB23**	**HXB26**	**Ratio**	**p-value**	**HXB23**	**HXB26**	**Ratio**	**p-value**	**Match Direction**

Afap1l1	6.44	6.69	0.84	0.0344	1.22	1.58	0.78	0.0199	Yes

Cckbr	7.92	8.31	0.76	0.0063	2.25	2.84	0.66	0.0110	Yes

P2rx4	8.45	7.52	1.90	0.0122	2.53	1.50	2.04	<.0001	Yes

Tnk2	8.80	8.68	1.09	0.3473	4.17	3.59	1.50	0.0214	Yes

Tubb6	6.92	7.00	0.94	0.5960	0.30	0.69	0.76	0.0400	Yes

Ptpre	7.70	7.63	1.05	0.2589	1.93	0.77	2.23	<.0001	Yes

Mogat2	5.26	5.10	1.12	0.3124	0.63	-0.63	2.39	0.0005	Yes

Nek3	6.05	5.90	1.10	0.1681	-0.36	-0.54	1.13	0.0388	Yes

Tmem2	7.25	7.32	0.95	0.3860	2.47	2.82	0.78	0.0086	Yes

Mc4r	6.01	5.82	0.87	0.0360	-0.39	-0.26	0.91	0.2058	Yes

F2rl1	6.16	6.26	0.94	0.3674	-2.10	-1.72	0.77	0.3943	Yes

Abat	10.52	10.35	1.13	0.0669	4.71	4.66	1.04	0.4464	Yes

Mboat2	8.73	8.46	1.21	0.0797	0.95	1.31	0.78	0.0051	No

Coq7	8.22	8.02	1.15	0.2095	2.60	3.76	0.45	<.0001	No

Dynlrb1	11.27	11.23	1.03	0.7064	5.96	6.00	0.97	0.4726	No

Tpst1	7.70	7.56	1.10	0.1642	3.12	3.77	0.64	0.0004	No

Fgfr2	9.44	9.41	1.02	0.7038	-1.10	4.25	0.02	<.0001	No

Pop5	7.53	7.55	0.98	0.9056	2.58	2.45	1.09	0.1509	No

Rab35	8.91	8.87	1.03	0.7719	4.18	4.28	0.93	0.0107	No

Spire1	9.78	9.74	1.03	0.3554	1.50	3.26	0.30	<.0001	No

Expression levels of the identified candidate genes were determined in brains (n = 5 per strain) of rats from two of the RI strains, HXB RI 26 (high alcohol consumption) and HXB RI 23 (low alcohol consumption) using CodeLink Rat Whole Genome Bioarrays and Affymetrix GeneChip Rat Gene 1.0ST arrays. These strains were chosen because the expression levels of all candidate genes (on CodeLink arrays) that were significantly correlated with alcohol consumption when all the RI strains were analyzed, showed an appropriate direction of differential expression in these two strains compared to the correlation analysis, even if the difference in expression between these 2 particular strains was not statistically significant. Details on analysis, normalization and statistical comparisons are provided in the text. Values represent mean of the log base 2 transformed expression data. "Ratio" is the HXB23/HXB26 ratio of the unlogged intensity values. P values are from the two-sample t-test, and "match direction" refers to the comparison between the two array experiments.

### Human Association Study

#### Outcome

Of the original 606 subjects in the WHO/ISBRA data from Montreal, 545 were self-reported Caucasian. Alcohol consumption in the Montreal population ranged from abstainers (0 g/kg/day) to heavy drinkers (maximum, 8.4 g/kg/day; see Additional file 1, Figure S1). Likewise, in the Sydney population, 242 of the original 285 subjects were Caucasian and alcohol consumption ranged from abstainers (0 g/kg/day) to heavy drinkers (maximum, 7.3 g/kg/day; see Additional file 1, Figure S1). Other covariates, and demographic characteristics, were comparable between the two populations with the exception of gender because only male subjects were recruited in Sydney (Additional file 1, Tables S2 and S3). For the purpose of comparability, we report results from only the male Caucasian subjects (n = 280) from the Montreal population.

#### Quality Control

Out of the 1,350 gene-related SNPs, 98 SNPs were not genotyped in any of the Montreal subjects (98 in Sydney subjects) and 88 were not informative across the Montreal sample (49 in the Sydney sample). Further evaluation of the remaining SNPs resulted in 121 SNPs being eliminated because they were not in Hardy-Weinberg equilibrium (HWE) in the Montreal sample (156 SNPs in the Sydney sample) (FDR<0.01). An additional 203 SNPs were eliminated in the Montreal sample (203 in Sydney) that had a minor allele frequency (MAF) that was less than 5% (46 SNPs in the Montreal population (79 in Sydney) did not meet HWE and had a MAF<5%). In addition, 110 subjects from Montreal and 61 subjects from Sydney were eliminated for having a call rate that was less than 80%, based on the criteria described in Hodgkinson et al. [14]. These exclusion criteria left 840 SNPs on 435 subjects for analysis in the Montreal population (220 males) and 845 SNPs on 181 subjects in the Sydney population.

#### Population Stratification

There was no evidence for population stratification at each site with respect to the outcome, i.e., alcohol consumption (chi-square = 344, df = 352, *P*-value = 0.62). However, there was substantial evidence for genetic difference between the population gathered in Sydney vs the population from Montreal (chi-square = 318, df = 176, *P*-value = 3.1 × 10^-10^). Because there was such strong evidence for genetic differences between the populations, the two populations were analyzed separately.

#### Genetic and Phenotypic Associations with Alcohol Consumption

Table 3 shows the SNPs that were significantly associated with alcohol consumption in the univariate analysis. In the Montreal population, SNPs representing two unique genes (GAD1, MPDZ) were significantly associated with alcohol consumption. Although the significant SNP in MPDZ only reached statistical significance in the dataset that combined both males and females, its FDR was 0.09 in Montreal males. For consistency, all further analyses in the Montreal population were only conducted on male subjects. In the Sydney population, SNPs representing four unique genes (CHRM5, GABRB2, MAPK1, PPP1R1B) were associated with alcohol consumption.

**Table 3:** Genetic (SNP) Associations with Alcohol Consumption: WHO/ISBRA Subjects

**refSNP ID**	**Location Within Gene**	**Chr**	**Basepair**	**minor allele/major allele**^**a**^	**MAF (Males/Females)**	**unadjusted p-value**	**FDR**
**MONTREAL**							

rs2241165	intronic region of GAD1	2	171386625	C/T	0.25/0.23	0.0001	0.0400

rs701492	intronic or downstream region of GAD1	2	171410726	T/C	0.31/0.31	<0.0001	0.0018

rs7578661	intronic region of GAD1	2	171423379	G/C	0.31/0.31	<0.0001	0.0018

rs1389752	intronic region of MPDZ	9	13225287	A/T	0.14/0.15	<0.0001^b^	0.0130^b^

**SYDNEY**							

rs8030094	intronic region of CHRM5	15	32124174	A/G	0.18/NA	0.0002	0.0326

rs10051667	intronic region of GABRB2	5	160830906	C/T	0.09/NA	<0.0001	0.0110

rs9607272	intronic region of MAPK1 and TUBA8^c^	22	20466398	G/T	0.19/NA	<0.0001	0.0062

rs879606	upstream region of PPP1R1B	17	35035375	A/G	0.13/NA	<0.0001	0.0062

Significant results from univariate association analysis with alcohol consumption. Shown are the eight SNPs that showed a significant (FDR<0.05) association with alcohol consumption (g/kg/day) in a genotype model.

^a^Minor and major allele designation is according to Ensembl release 50.

^b^This SNP was significant when both genders are combined; in male subjects, the FDR for this SNP was 0.09. All other SNPs shown were significant when males only were tested.

^c^The localization of this SNP is ambiguous. Its presence in MAPK1 and the overlapping gene, TUBA8 (tubulin alpha 8) is of particular interest, given the findings of differences in expression of cytoskeletal genes in the HXB/BXH rat strains.

We determined the haplotype block that contained each significant SNP. For GAD1 in the Montreal sample, three SNPs significantly associated with alcohol consumption were located within the same known haplotype block (Additional file 1, Figure S2). Haplotypes and individual SNPs were evaluated for their association with alcohol consumption to determine the best fit model for each gene.

The final genetic multivariate model for Montreal male subjects included the recessive effect of haplotype 2 (see Additional file 1, Table S4) in MPDZ and the SNP genotype effect for rs701492 in GAD1 (Table 4). In combination, these two genetic factors explained 16% of the total variance in alcohol consumption. For the subjects from Sydney, three of the candidate genes were represented by SNPs in the final genetic multivariate model (Table 4). For the fourth candidate gene, MAPK1, the best fit to the data was a model with both an additive and dominant component for haplotype 3 (see Additional file 1, Table S4); however, this gene was not included in the final genetic multivariate model.

**Table 4:** Multivariate Models with Covariates for Alcohol Consumption

**MONTREAL (r^**2 **^= 0.62)**				
**Effect**	**Estimate**	**Standard Error**	**t-statistic**	**p-value**
Intercept	-0.59	0.13	-4.59	<.0001
recessive haplotype 2 - MPDZ	1.02	0.24	4.17	<.0001
rs701492 - GAD1 (AA)	0.70	0.10	6.94	<.0001
rs701492 - GAD1 (AB)	-0.03	0.07	-0.42	0.6714
rs701492 - GAD1 (BB)	referent			
Age	0.01	0.00	4.50	<.0001
Alcohol dependence in past year	1.04	0.10	10.73	<.0001
Alcohol abuse in past year	0.73	0.10	7.67	<.0001
Family history of depression (1st degree relative)	0.50	0.09	5.45	<.0001
Familial depression and alcohol dependence*	-0.85	0.14	-6.09	<.0001
Used medication other than antidepressants in last month	0.26	0.07	3.91	0.0001
				
**SYDNEY (r^**2 **^= 0.62)**				
				
**Effect**	**Estimate**	**Standard Error**	**t-statistic**	**p-value**
Intercept	0.24	0.06	4.12	<.0001
CHRM5 (recessive)	2.10	0.35	5.95	<.0001
GABRB2 (dominant)	3.03	0.43	7.00	<.0001
PPP1R1B (recessive)	2.97	0.32	9.22	<.0001
Alcohol dependence in past year	0.58	0.11	5.06	<.0001
Alcohol abuse in past year	0.58	0.11	5.14	<.0001

Shown are the coefficients associated with the final multivariate models with covariates for alcohol consumption as outcome. Final models were chosen using a backward selection process with an exit criterion of p > 0.001.

* Familial depression is defined as major depression in the subject and in a first-degree relative.

We also analyzed the relationship of phenotypic characteristics with alcohol consumption levels by males in Sydney and Montreal. As expected, our data showed that alcohol consumption was positively correlated with alcohol dependence in both populations (Additional file 1, Figure S3). The multivariate model with covariates for the Sydney population included alcohol abuse and the diagnosis of alcohol dependence (DSM-IV) (Table 4). In the population from Montreal, the multivariate model with covariates included a diagnosis of major depression and a family history of depression, as well as alcohol abuse and dependence (Table 4). In both populations the models including genetic and phenotypic characteristics accounted for 62% of the variance in alcohol consumption. Our further analysis indicated that none of the genotypes or haplotypes that were found to be significantly associated with alcohol consumption were significantly associated with alcohol abuse or alcohol dependence in either population (FDR >0.05). In fact, we found none of the SNPs represented on the "addiction array" to be significantly associated with DSM-IV alcohol dependence in either the Sydney or Montreal populations (FDR >0.05). A caveat to keep in mind is that the sample sizes may not have provided enough power to allow us to detect associations using the binary outcomes of alcohol dependence or abuse.

## Discussion

The reasons for differential intake of ethanol by individuals in a population, be it humans or other animals, have been the subject of an immense amount of research. The current dogma posits that both environmental and genetic factors contribute to individual differences in alcohol consumption [32,33]. What is sometimes confusing about the literature on alcohol consumption is the lack of discrimination between alcohol consumption in non-dependent vs alcohol-dependent individuals [34]. The DSM IV and ICD-10 criteria for alcohol dependence focus intensively on dichotomizing alcohol drinking behavior in dependent and nondependent individuals [35,36] and several recent reviews by authors of this current work [37], and others [9], have detailed evidence and hypotheses regarding the progression of events which transform the non-dependent alcohol intake phenotype to the alcohol-dependent alcohol intake phenotype.

Epidemiologic studies do indicate that the higher the levels of alcohol intake by an individual, the higher is the propensity for an individual to become alcohol dependent [38]. In this regard, high levels of alcohol intake become a risk factor for transition to alcohol dependence, but there is no *a priori *reason to assume that the genetic factors that may be responsible for modulating an individual's non-dependent levels of alcohol consumption are the same factors that predispose or protect an individual from becoming dependent. In other words, a genetic relationship between the propensity for high alcohol intake and the propensity for becoming alcohol dependent has not been demonstrated. In fact, one can interpret some of the data collected with mice to show a dissociation between a propensity for high alcohol preference and propensity for physical dependence. C57BL/6 mice are a genetically inbred strain that has been demonstrated to have one of the highest levels of alcohol preference, but these mice have a low propensity to develop alcohol dependence (exemplified by signs of CNS withdrawal hyperexcitability) [2,39,40]. It should thus be emphasized that the phenotype we are investigating in this paper is non-dependent alcohol intake.

What emanates as a conclusion from our genetical genomic/phenomic approach for the search for "candidate genes" that influence non-dependent alcohol intake is that a group of genes/gene products that can be linked to systems which, in the rat (and human), control appetite and satiety, play an important role in variations in the non-dependent alcohol intake phenotype (see 5). It is of interest that our earlier work, which involved a meta-analysis of data from recombinant inbred mice, selectively bred mice and a large panel of inbred mice, indicated that orosensory systems in the mice are critically involved in alcohol selection in a two-bottle choice experimental design [2]. Our two studies, taken together with a number of prior studies [41], indicate that alcohol selection by rodents involves the sensory and caloric information transduction systems dealing with the recognition, and the satiating and rewarding properties of foodstuffs. Ethanol, unlike other psychoactive drugs, has significant caloric value (seven cal/gm) and these calories have been clearly shown to have relevance to an organism's energy status [42].

**Table 5:** Function of Replicated Candidate Gene Products

**Correlation with Alcohol Consumption**	**Gene Symbol**	**Gene Name and Description**
+	*Mc4r*	Melanocortin 4 receptor; activation by melanocortin (MSH) decreases caloric intake. Mc4r localized on GABA neurons. Mc4r agonist reduced ethanol consumption in association with food consumption [45,65,116].

+	*Cckbr*	Cholecystokinin 2 receptor; Cholecystokinin (CCK) is localized in some GABA interneurons, and activation of Cck2r can facilitate GABA release. CCK is a satiety hormone. Cck2r null mutants show low alcohol consumption [60,95,117].

-	*Mogat2* *= MGAT2*	Monoacylglycerol O-acyltransferase 2; esterase affecting mono- and di-acylglycerol.

-	*Abat*	4-aminobutyrate aminotransferase (GABA aminotransferase); GABA degrading enzyme.

-	*P2rx4*	P2x purinoceptor 4; located on GABA neuron terminals in VTA and is a cation channel for Ca^2+^. Enhances GABA release by presynaptic action. Inhibited by ethanol [118].

-	*Ptpre*	Receptor type protein tyrosine phosphatase epsilon; inhibits voltage-gated K^+ ^channels on GABA interneurons [62].

+	*Afap1l1*	Actin filament associated protein; interacts with dynamin, involved in endocytosis.

+	*Tubb6*	Tubulin B6; cytoplasmic dynein interacts with tubulin, involved in microtubule movement [79].

-	*Tnk2*	Protein tyrosine kinase Ack; Cdc42-associated kinase, located pre and post synaptically; influences endocytosis [72].

+	*F2rl1*	Protease-activated receptor 2(PAR2); activates PI3-kinase, activation of Cdc42 required. Increases Ca^2+ ^signaling. In Glu and GABA neurons [75,76].

**Figure 3:**
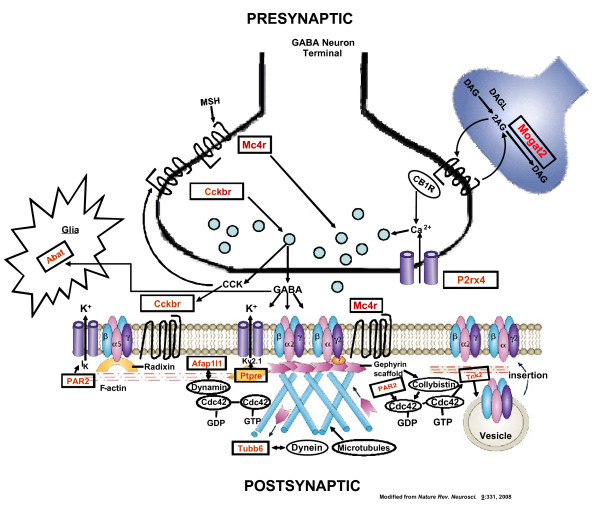
**Candidate Genes from HXB/BXH RI Rat Strain Microarray Analysis Proposed for Presynaptic GABA Neuron Terminal and Postsynaptic Neuron**. The identified candidate genes are indicated by rectangles. Presynaptic gene products are suggested to modulate GABA release, and postsynaptic gene products affect GABA_A _receptor localization and trafficking in VTA dopaminergic neurons.

Figure 3 illustrates that most of the candidate genes that we identified can be related within a neurobiological pathway that was constructed based on analysis of the literature. Many of the candidate genes identified in this study code for products (protein) that have been implicated in regulation of feeding and energy metabolism. For example, the cholecystokinin 2 (CCK2) receptor is expressed in hypothalamic regions such as the paraventricular nucleus (PVN), which contains the circuitry necessary for maintaining energy homeostasis (food intake vs energy expenditure). CCK released from the gut acts as a "satiety hormone" [43], and can activate neurons in various hypothalamic regions [44]. The melanocortin 4 receptor (Mc4r) is also expressed in the hypothalamic regions that regulate energy metabolism, and activation of these receptors by the endogenous agonist, MSH, also inhibits food intake. It has been suggested that these actions involve changes in GABA release from hypothalamic interneurons [45,46]. Both *Mc4r *and *CCK2R *are expressed at higher levels in animals that consume higher amounts of alcohol, suggesting that these animals may have an inherent propensity for reduced food intake and lower energy expenditure.

However, animals and humans ingest food not only for nourishment, but also for the rewarding properties of food, and motivational mechanisms are important for generating responses needed for food-seeking and consumption behavior. The neuronal pathways and neurotransmitter substances that regulate food intake for energy homeostasis, and for the rewarding properties of food, are intimately connected but not identical. Particularly relevant to our current studies are the hypothalamic nuclei with connections to the ventral tegmental area (VTA) that generate and carry information on hunger and satiety, and initiate the cascade of events resulting in "wanting/liking" food or other "rewards" [47,48]. Much of the hypothalamic input to the VTA which provides information on the energy status of the mammalian organism is initiated in the arcuate nucleus of the hypothalamus and transmitted via the lateral hypothalamus to the VTA dopaminergic neurons. The transmitter substance that has been associated with stimulatory input from the lateral hypothalamus is orexin, which activates the dopaminergic neurons of the VTA [49]. GABA neurons provide the major direct inhibitory input to the dopaminergic neurons of the VTA, but GABA neurons arising in the arcuate nucleus also provide inhibitory input to the orexin neurons residing in the lateral hypothalamus [49]. Thus, GABAergic activity related to food/energy requirements can generate inhibitory influences on the VTA dopaminergic neuron firing either directly or through inhibition of orexin signaling to the VTA.

The products of many of the candidate genes identified in this study can affect GABAergic neuronal activity. One of the most studied systems in this regard, and one suggested to link the homeostatic and reward pathways associated with food intake [50], is the endocannabinoid system. The endogenous agonists anandamide and 2-AG (2-arachidonoyl glycerol) act primarily through presynaptic cannabinoid 1 (CB1) receptors to inhibit GABA release [51]. The endocannabinoid system in the hypothalamus has been implicated in regulation of both food and ethanol intake [52,53]. 2-AG can act as an anterograde or retrograde messenger to inhibit GABA release [54], and can then be inactivated by hydrolysis by monoacylglycerol lipase or fatty acid amide hydrolase [55]. More recently, however, another group of enzymes has been proposed to inactivate 2-AG, not by hydrolysis, but by conversion of 2-AG to diacylglycerol. The enzyme carrying out this reaction is the product of *Mogat2 *(also referred to as *Mgat2*))[56]. *Mogat2 *mRNA was found to be expressed at lower levels in the higher alcohol-drinking rats, possibly resulting in a reduced rate of metabolism of 2-AG, leading to greater inhibition of GABA release.

The cholecystokinin 2 (CCK2) receptor is expressed not only in the hypothalamus, but also in other brain regions, such as the VTA or amygdala, where it is localized presynaptically on GABA neurons [57,58]. The activation of the CCK2 receptor has been shown to promote action potential-induced GABA release in certain brain areas (e.g., hippocampus) [59], but more recent data suggest that the initial increase in GABA release produced by CCK via the CCK2 receptors is followed by a reduction in GABA release [60]. Our findings of higher levels of the mRNA for the CCK2 receptor in high alcohol consuming rats could be construed as another differential means of modulating GABA release in low and high-drinking rats. Another candidate gene product, the P2X_4 _purinergic receptor (the product of *P2rx4*), is also localized presynaptically on GABA neuron terminals [61]. Activation of P2X_4 _receptors on GABA terminals may increase GABA release [61]. Since P2X_4 _receptor mRNA is expressed at lower levels in the high alcohol-consuming animals, this again would point to diminished levels of GABA release in such rats.

Another identified candidate gene may further regulate the excitability of GABA neurons and GABA release. *Ptpre *encodes the receptor type protein tyrosine phosphatase epsilon, which has been reported to dephosphorylate and decrease the activity of the voltage-gated potassium channel, Kv2.1 [62]. The Kv2.1 channel is found on somata and proximal dendrites of inhibitory (GABAergic) interneurons [see [62]]. The expression of *Ptpre *is lower in animals that consume higher amounts of alcohol, and if this results in a lesser level of phosphatase activity and higher levels of the channel activity, one would expect lower excitability of GABAergic interneurons in these animals.

On the other hand, another of the candidate genes, Abat, codes for the enzyme 4-aminobutyrate (GABA) aminotransferase, which degrades GABA. This gene is expressed at lower levels in animals with higher ethanol consumption, and this situation could contribute to enhanced GABA levels in GABAergic synapses in the high alcohol-consuming rats.

The effects on GABAergic activity described above could contribute to a decreased hypothalamic GABAergic inhibitory effect on VTA dopaminergic activity in the high alcohol-consuming rats. Furthermore, the products of a number of the candidate genes can more directly affect the activity of midbrain dopamine neurons. MSH, administered into the VTA, increases dopamine release in the nucleus accumbens via actions on VTA melanocortin 4 receptors [63]. Interestingly, administration of a melanocortin 4 receptor agonist to mice (icv) reduced alcohol drinking [64], and administration of a melanocortin 3/4 receptor agonist to alcohol-preferring rats reduced alcohol intake in association with reduced food intake [65]. The CCK2 receptor is also found in the VTA [57] and likely responds to CCK release from GABAergic neurons synapsing directly with dopaminergic cell bodies in this region. Administration of CCK into the VTA results in increased dopamine release in the nucleus accumbens, mediated by activation of the CCK2 receptors in the VTA [57,66]. Taken together with the inherent differences in expression of genes whose products influence GABA release, the greater expression of the CCK2 and MC4 receptors, if it occurs in the VTA of the high alcohol consuming animals, generates a picture of lower GABAergic inhibitory tone and an increased propensity for direct dopaminergic neuron activation in the VTA by MSH and CCK.

Recent work has shown that the quantities of ethanol consumed by rats in a two-bottle choice paradigm, can, in fact, enhance the electrophysiological activity of VTA dopaminergic neurons [67]. What is unclear about such studies is whether these effects of ethanol are direct or indirect, through modulation of transmitters influencing dopamine neuron firing. For example, acutely, ethanol inhibits the function of the P2X_4 _receptor [68], and this action may reduce the presynaptic function of the P2X_4 _receptor and reduce GABA release, thus disinhibiting dopaminergic activity. Xiao and Ye [69] have also reported that ethanol can acutely inhibit GABAergic neuron activity in the VTA via other indirect mechanisms, while enhancing such activity in other brain areas. These actions of ethanol in the VTA would tend to disinhibit dopaminergic neuron activity and synergize with the inherently lower GABAergic tone suggested by our studies.

Another intriguing aspect of our candidate gene list is the presence of a number of genes coding for products that can affect postsynaptic aspects of GABA receptor trafficking. These candidate genes and gene products are related to rho GTPases, which affect the cytoskeleton, membrane trafficking and cell adhesion through their regulation of actin dynamics. For example, Cdc42 is a member of the rho GTPase family that can stimulate actin polymerization, affecting specific steps of vesicle trafficking such as those involved in endocytosis [70]. Cdc42 can be activated by a protein called collybistin, which is a gephyrin-binding protein [70]. Gephyrin is a scaffolding protein for GABA_A _and glycine receptors [71], and these interactions provide support for a specific role for Cdc42 in clustering and trafficking of GABA_A _receptors.

Two of the candidate genes that we identified are Cdc42-associated proteins. *Tnk2 *codes for a cytosolic non-receptor tyrosine kinase, also known as Ack (activated Cdc42-associated tyrosine kinase). This kinase has been identified as a downstream effector of Cdc42 which is important for regulation of receptor degradation via endocytic mechanisms [72]. It is widely expressed in brain, including various nuclei of the mesencephalon [73]. *F2rl1 *codes for a protease-activated receptor known as Par2. This G protein-coupled receptor is also widely expressed in brain, including relatively high expression in amygdala, and is found in the soma and dendrites of GABAergic and glutamatergic neurons [74,75]. Par2 agonists activate several cell signaling molecules, including phosphoinositide 3-kinase. This latter activity requires Cdc42 activation by Par2 [76]. The negative association of the expression of Ack with alcohol preference, and the positive association of Par2 expression, could enhance the endocytic recycling process associated with Cdc42-mediated actin polymerization in the animals that consume higher amounts of alcohol, leading to more rapid GABA receptor desensitization.

The products of some other candidate genes are associated with actin organization and microtubule activity, which is also important for the trafficking of receptors and other proteins. Afap1l1 codes for an actin filament associated protein. The expression of this gene is positively correlated with alcohol consumption. Microtubules are involved not only in receptor transport, but also in the movement of gephyrin to and from the synaptic plasma membrane [77]. Dynein is a microtubule motor that participates in axonal transport of neurofilaments [78], and that interacts with beta tubulin [79], the protein product of another of the candidate genes, *Tubb6*. The expression of all of the above-mentioned candidate genes is associated positively with alcohol consumption in the RI rat strains, supporting the postulate that there may be enhanced GABA_A _receptor trafficking (endocytosis), in the brains of animals that have a propensity to consume more alcohol.

Overall, our analysis of the functional pathways defined by the identification of the candidate genes in the HXB/BXH rat strains focuses attention on GABAergic and dopaminergic activity that may set a tone that predisposes to (or against) voluntary alcohol consumption. We are proposing that, in rats, a lower inherent GABAergic tone generated by reduction in presynaptic release and a more responsive GABA receptor desensitization system predisposes to higher alcohol consumption in a free choice experimental paradigm. There are numerous studies showing that administration of agonists or antagonists of GABA or dopamine receptors can alter alcohol consumption or self-administration by rats [e.g., [80-90]].

Although we have focused attention on neuronal systems which mediate the animals' recognition of the energy status of the body and the rewarding properties of caloric substances, it should be clearly stated that the candidate genes we identified, and their products, do play important roles in other anatomically-defined neural systems. A case in point may be that dopaminergic neurons which innervate the dorsal striatum (cell bodies in the substantia nigra) may have as much to do with appetitive behavior as the systems we describe innervating the nucleus accumbens from the A10 nucleus (VTA) [91]. Another area of brain where a number of the products of the candidate genes that we identified interact is the amygdala. For instance, *Mc4r *is expressed in several nuclei of the amygdala and in the bed nucleus of the stria terminalis [92,93]. Studies with melanocortin 4 receptor null mutant mice showed that restoration of the receptor in a population of amygdala neurons reduced food intake [94], similar to the effect of activation of this receptor in hypothalamus. *Cckbr *is expressed in the amygdala as well, and CCK increases GABA release in amygdala via the CCK2 receptor [95]. Higher levels of GABA release in the amygdala may set a tone that predisposes to higher alcohol consumption, given the finding that delivery of GABA_A _receptor antagonists to the amygdala reduced ethanol self-administration [81]. The endocannabinoid system can also interact with the CCK system in the amygdala, as well as modulating GABA release in this area of brain. CB1 receptor agonists inhibit the release of CCK from GABAergic neurons and Chhatwal et al. [96] have shown that the effects of cannabinoids on the extinction of conditioned fear responses are mediated through CCK2 receptors. Clearly the neuronal systems that affect conditioned fear (anxiety) responses are also involved with appetitive and reward pathways [97,98].

The ultimate goal of genetic studies using animal models of alcohol consumption is to identify candidate genes that influence a human's level of alcohol consumption. It should be stressed at this point, that the phenotype we utilized for our genetic association studies with humans was also the quantitative measure of alcohol intake. This phenotype was chosen to allow proper comparison with our studies with the rats. We also performed a separate analysis of the genetic association of the phenotype of alcohol dependence defined by both DSM IV and ICD-10 criteria with the panel of 1,350 SNP marker included on the "Addiction Array" [14]. The polymorphisms that we found to be associated with the quantitative alcohol consumption phenotype in human populations, identified genes whose products are involved in the pathways determined from our studies of differential levels of alcohol intake by the panel of recombinant inbred rats. Particularly evident were components of the GABAergic signaling pathway, including the β2 subunit of the GABA_A _receptor in the Sydney population, and the GABA synthetic enzyme, GAD1, as well as the MPDZ protein, which can act as a scaffolding protein for the GABA_B _receptor [99], in the Montreal population. MPDZ has previously been linked to alcohol withdrawal seizure susceptibility in mice [100], but not with levels of alcohol intake. The other genes identified in the Sydney population have also been linked to mesolimbic dopaminergic activity. CHRM5, the M5 muscarinic cholinergic receptor subtype, localized on VTA dopamine neurons, is thought to contribute to tonic excitatory regulation of dopamine transmission [101]. As noted earlier, our studies do indicate that the products of the candidate genes predisposing high alcohol drinking by rats can be linked to the modulation of VTA dopamine neuron function. Additionally, muscarinic cholinergic receptors in the VTA have been shown to play an important role in alcohol selection by rats selectively bred (P rats) for high alcohol intake [102]. PPP1R1B, also known as DARPP-32, as well as MAP kinase (the product of MAPK1 gene), are downstream targets of dopamine D1 receptors in the medium spiny neurons of the shell of the nucleus accumbens and other brain areas receiving input from the VTA. Although the "Addiction Array" SNPs did not allow us to assess polymorphisms in a number of the candidate genes identified in our studies with rats (e.g., *Mc4r*), it has been demonstrated that activation of melanocortin 4 receptors in the nucleus accumbens could enhance dopamine D1 receptor-mediated cyclic AMP production [103], and modulate the enzymatic activity of DARPP-32. The *Mc4r *mRNA, which we found to be elevated in brains of the alcohol-preferring rats, is reported to be found in the nucleus accumbens, apparently in medium spiny neurons, as well as in the VTA and hypothalamus of humans [92,103].

The overall impression that is generated by cursorily examining the results of our studies with rats and humans, and our prior studies with mice, is that little evidence may have been produced to indicate that identical genes or gene products predispose free choice alcohol intake in rodents or humans. What may be missed is the fact that certain identical neurobiologic pathways have been identified in all of these investigations. In rodents, one can posit that neurobiologic systems that participate in sensing and transducing information about the rewarding or aversive properties of foodstuffs play an important role in oral consumption of ethanol in a free-choice paradigm. Polymorphisms in the loci of genes involved in such an appetitive pathway are also associated with quantitative measures of alcohol intake in humans [104,105]. The proposition that appetitive sensory systems and/or caloric qualities of ethanol contribute to ethanol drinking by rodents is not novel [41,106-108], but the identification of such a relationship by an unbiased genetical genomic/phenomic technique allows one to progress to examination of specific pathways and genetic mechanisms in future studies.

Our results also cast some light on the influence of genetic polymorphisms on levels of alcohol drinking vs alcohol dependence in humans. Epidemiologic studies suggest a strong correlation between levels of ethanol consumed and the diagnosis of alcohol dependence [38], and alcohol intake has been taken by some as a surrogate (endophenotype) for alcohol dependence [109]. In our study, the polymorphisms that we found to be associated with the level of alcohol consumption by humans continued to have an influence in the multivariate model, even when current alcohol dependence and alcohol abuse were accounted for. This result, and the lack of association of the candidate genes for levels of alcohol consumption, with alcohol dependence/abuse in the same individuals, suggest that the genetic factors that we identified as predisposing factors for alcohol consumption may have little direct influence on alcohol dependence or abuse, but would be important for generating the major risk factor for dependence (i.e., high levels of alcohol consumption).

A recent genome-wide association study of alcohol dependence in humans used genes identified by differential mRNA expression in alcohol-consuming rats as a means of attempting to generate more credence in the candidate genes with modest statistical support for association with alcohol dependence in the analysis of the human data [110]. Our studies would caution against such an approach because genetic determinants for alcohol consumption by animals or humans may not be identical with genetic determinants of ethanol dependence. This contention is also supported by QTL analysis of free-choice alcohol consumption [111] and alcohol dependence/withdrawal [112] in animals, and alcohol consumption/alcohol dependence in humans [113-115]. The most recent and instructive study in this regard is a study with humans performed by Hansell et al. [105]. This study utilized quantity/frequency measures of alcohol consumption as a phenotype for a linkage study and found two types of QTLs. The LOD scores for certain QTLs were diminished if the primarily high level alcohol consumers were utilized for analysis, and other QTLs were enhanced if primarily high level consumers were used. What was interesting is that the QTLs which were enhanced when individuals with lower levels of alcohol consumption were dropped from the analysis were those which, in other studies, were related to "alcoholism" or alcohol dependence. QTLs identified as significant and having maximal LOD scores using the full range of alcohol consumption in this population, were not in areas of the human genome previously associated with alcohol dependence. However, at least one such QTL was previously found by de Andrade et al. to be associated with the number of drinks (on average) consumed by the subjects collected in the COGA study [109].

## Conclusion

The genetical genomics approach, in combination with phenomics, is a powerful method for determination of candidate genes that contribute to the predisposition to alcohol consumption in rats. Informatics-based analysis of the function of candidate gene products led to the consideration that GABAergic function, and particularly GABA release modulated by peptidergic, purinergic and endogenous cannabinoid systems, as well as GABA receptor trafficking, are important components of the genetic/biochemical pathways that contribute to alcohol drinking. Our results highlight the need to identify neuronal signaling networks based on candidate genes, rather than focusing on individual genes and gene products, when attempting to understand the genetic basis of complex behavioral traits. The comparison of rat and human genetic contributors to the trait of alcohol consumption suggested that one can extrapolate from pathways - not necessarily specific genes - found in animals to begin to elucidate cross-species similarities in the genetic basis of behavior. Our results also emphasize the importance of carefully defining phenotypes for genetical genomic approaches; in this case, although high levels of alcohol drinking are phenotypically correlated with alcohol dependence, the genetic factors that contribute to the full range of alcohol consumption versus alcohol dependence in humans are distinct.

## Authors' contributions

BT planned and supervised all experiments, analyzed data and drafted the manuscript. LS carried out eQTL and bQTL analyses, correlation and all statistical analyses. MPrintz provided data and tissue from the HXB/BXH rats, participated in alcohol consumption studies, and analyzed alcohol consumption data. PF participated in HXB/BXH rat alcohol consumption experiments and analyzed data. CH performed studies using the addiction array. DG supervised addiction array studies and analyzed data. GK conceived, organized and supervised the HXB/BXH alcohol consumption studies. HNR organized and supervised the HXB/BXH rat alcohol consumption studies and analyzed data. KK performed transcription factor and transcription factor binding site analysis. RLB provided data and tissue from P and NP rats. NH performed genotyping of HXB/BXH RI strains. MH performed genotyping of HXB/BXH RI strains. MPravenec provided tissue from HXB/BXH rats. JM provided genotype data from HXB/BXH RI rats. LL recruited subjects from Montreal and provided data and DNA. MD supervised studies and recruited subjects in Montreal. KMC recruited subjects in Australia and provided data and DNA. JW supervised human studies and recruited subjects in Australia. JS recruited subjects in Australia and analyzed data. BG created questionnaire for WHO/ISBRA study and generated algorithms for data analysis. PLH analyzed microarray data and drafted manuscript. WHO/ISBRA Investigators are included as authors on all studies using data from the WHO/ISBRA study, by formal agreement.

## Supplementary Material

Additional file 1 **Supplemental Methods and Results**. Table S1: Identification of transcription factors in eQTL on chromosome 6. Table S2: Distribution of covariates used for multivariate model with human subjects. Table S3: Demographic characteristics of human subjects. Table S4: Haplotype frequencies and association with alcohol consumption in human subjects. Figure S1: Alcohol consumption levels in human subjects. Figure S2: Haplotype block assessments. Figure S3: Relationship between alcohol consumption and alcohol dependence in human subjects. Click here for file
