# Supplementary material for: Genetical genomic determinants of alcohol consumption in rats and humans
Source: BMC Biol. 2009 Oct 27;7:70. doi: 10.1186/1741-7007-7-70 (PMC2777866; doi:10.1186/1741-7007-7-70)
Supplement: Additional file 1 — Supplemental Methods and Results. Table S1: Identification of transcription factors in eQTL on chromosome 6. Table S2: Distribution of covariates used for multivariate model with human subjects. Table S3: Demographic characteristics of human subjects. Table S4: Haplotype frequencies and association with alcohol consumption in human subjects. Figure S1: Alcohol consumption levels in human subjects. Figure S2: Haplotype block assessments. Figure S3: Relationship between alcohol consumption and alcohol dependence in human subjects. [file 1741-7007-7-70-S1.DOC]

**Supplemental Methods and Results**

Identification of Transcription Factors in eQTL on Chromosome 6.

Using the Ensembl 50 database (rat genome version 3.4) a window around the eQTL hotspot on chromosome 6 (20 - 45 MB) was examined for the location of transcription factors. There were 18 genes identified as transcription factors according to "transcription" or "DNA binding" related Gene Ontology ([www.geneontology.org](../www.geneontology.org)) terms obtained from Ensembl on each gene. Seventeen of those 18 also had mouse orthologs according to Ensembl 50 (mouse genome version NCBI37) (Table 1).

| **Table 1** | | | | |
| --- | --- | --- | --- | --- |
| **Ensembl Gene ID** | **Description** | **ID** | **Mouse Ensembl Gene ID** | **Mouse External ID** |
| [ENSRNOG00000000216](http://www.ensembl.org/Rattus_norvegicus/geneview?gene=ENSRNOG00000000216) | transcription factor CP2-like 2 [Source:RefSeq_peptide;Acc:NP_001032431] | Tcfcp2l2 | [ENSMUSG00000020653](http://www.ensembl.org/Mus_musculus/geneview?gene=ENSMUSG00000020653) | MGI:2653358 |
| [ENSRNOG00000004068](http://www.ensembl.org/Rattus_norvegicus/geneview?gene=ENSRNOG00000004068) | Nuclear receptor coactivator 1 (EC 2.3.1.48) (NCoA-1) (Steroid receptor coactivator 1) (SRC-1) (Nuclear receptor coactivator protein 1) (mNRC-1). [Source:Uniprot/SWISSPROT;Acc:P70365] | MGI:1276523 | [ENSMUSG00000020647](http://www.ensembl.org/Mus_musculus/geneview?gene=ENSMUSG00000020647) | MGI:1276523 |
| [ENSRNOG00000004449](http://www.ensembl.org/Rattus_norvegicus/geneview?gene=ENSRNOG00000004449) | Transcription factor E2F6 (E2F-6) (E2F-binding site-modulating activity protein) (EMA). [Source:Uniprot/SWISSPROT;Acc:O54917] | MGI:1354159 | [ENSMUSG00000057469](http://www.ensembl.org/Mus_musculus/geneview?gene=ENSMUSG00000057469) | MGI:1354159 |
| [ENSRNOG00000004506](http://www.ensembl.org/Rattus_norvegicus/geneview?gene=ENSRNOG00000004506) |  | LOC690450 | [ENSMUSG00000059669](http://www.ensembl.org/Mus_musculus/geneview?gene=ENSMUSG00000059669) | MGI:109577 |
| [ENSRNOG00000004667](http://www.ensembl.org/Rattus_norvegicus/geneview?gene=ENSRNOG00000004667) |  | NP_001100187.1 | [ENSMUSG00000051235](http://www.ensembl.org/Mus_musculus/geneview?gene=ENSMUSG00000051235) | MGI:2443149 |
| [ENSRNOG00000004694](http://www.ensembl.org/Rattus_norvegicus/geneview?gene=ENSRNOG00000004694) |  |  |  |  |
| [ENSRNOG00000005938](http://www.ensembl.org/Rattus_norvegicus/geneview?gene=ENSRNOG00000005938) | Protein Mpv17. [Source:Uniprot/SWISSPROT;Acc:Q5BK62] | Mpv17l_predicted | [ENSMUSG00000029144](http://www.ensembl.org/Mus_musculus/geneview?gene=ENSMUSG00000029144) | MGI:1919002 |
| [ENSRNOG00000006090](http://www.ensembl.org/Rattus_norvegicus/geneview?gene=ENSRNOG00000006090) | Urocortin precursor (Corticotensin). [Source:Uniprot/SWISSPROT;Acc:P55090] | Ucn | [ENSMUSG00000038676](http://www.ensembl.org/Mus_musculus/geneview?gene=ENSMUSG00000038676) | MGI:1276123 |
| [ENSRNOG00000006308](http://www.ensembl.org/Rattus_norvegicus/geneview?gene=ENSRNOG00000006308) | N-myc proto-oncogene protein. [Source:Uniprot/SWISSPROT;Acc:Q63379] | Mycn | [ENSMUSG00000037169](http://www.ensembl.org/Mus_musculus/geneview?gene=ENSMUSG00000037169) | MGI:97357 |
| [ENSRNOG00000007141](http://www.ensembl.org/Rattus_norvegicus/geneview?gene=ENSRNOG00000007141) | Prolactin regulatory element-binding protein (Mammalian guanine nucleotide exchange factor mSec12). [Source:Uniprot/SWISSPROT;Acc:Q9WTV0] | PREB_RAT | [ENSMUSG00000045302](http://www.ensembl.org/Mus_musculus/geneview?gene=ENSMUSG00000045302) | MGI:1355326 |
| [ENSRNOG00000007237](http://www.ensembl.org/Rattus_norvegicus/geneview?gene=ENSRNOG00000007237) | DNA-binding protein inhibitor ID-2 (Inhibitor of DNA binding 2). [Source:Uniprot/SWISSPROT;Acc:P41137] | Id2 | [ENSMUSG00000020644](http://www.ensembl.org/Mus_musculus/geneview?gene=ENSMUSG00000020644) | MGI:96397 |
| [ENSRNOG00000009967](http://www.ensembl.org/Rattus_norvegicus/geneview?gene=ENSRNOG00000009967) | Otoferlin (Fer-1-like protein 2) (Fragment). [Source:Uniprot/SWISSPROT;Acc:Q9ERC5] | Otof | [ENSMUSG00000062372](http://www.ensembl.org/Mus_musculus/geneview?gene=ENSMUSG00000062372) | MGI:1891247 |
| [ENSRNOG00000025736](http://www.ensembl.org/Rattus_norvegicus/geneview?gene=ENSRNOG00000025736) |  | LOC688600 | [ENSMUSG00000006642](http://www.ensembl.org/Mus_musculus/geneview?gene=ENSMUSG00000006642) | MGI:1934960 |
| [ENSRNOG00000026578](http://www.ensembl.org/Rattus_norvegicus/geneview?gene=ENSRNOG00000026578) | DnaJ (Hsp40) homolog, subfamily C, member 5 gamma [Source:RefSeq_peptide;Acc:NP_001013260] Dnajc5g GO:0031072 heat shock protein binding |  | [ENSMUSG00000053856](http://www.ensembl.org/Mus_musculus/geneview?gene=ENSMUSG00000053856) | MGI:3045263 |
| [ENSRNOG00000026649](http://www.ensembl.org/Rattus_norvegicus/geneview?gene=ENSRNOG00000026649) | DNA (cytosine-5)-methyltransferase 3A (EC 2.1.1.37) (Dnmt3a). [Source:Uniprot/SWISSPROT;Acc:Q1LZ53] | Dnmt3a | [ENSMUSG00000020661](http://www.ensembl.org/Mus_musculus/geneview?gene=ENSMUSG00000020661) | MGI:1261827 |
| [ENSRNOG00000028518](http://www.ensembl.org/Rattus_norvegicus/geneview?gene=ENSRNOG00000028518) | similar to mesogenin 1 (LOC689864), mRNA [Source:RefSeq_dna;Acc:NM_001109551] | LOC686162 | [ENSMUSG00000047002](http://www.ensembl.org/Mus_musculus/geneview?gene=ENSMUSG00000047002) | MGI:1860483 |
| [ENSRNOG00000032178](http://www.ensembl.org/Rattus_norvegicus/geneview?gene=ENSRNOG00000032178) | similar to centromere protein A (predicted) (RGD1563607_predicted), mRNA [Source:RefSeq_dna;Acc:NM_001106711] NP_001100181.1 GO:0003677 DNA binding |  | [ENSMUSG00000029177](http://www.ensembl.org/Mus_musculus/geneview?gene=ENSMUSG00000029177) | MGI:88375 |
| [ENSRNOG00000039902](http://www.ensembl.org/Rattus_norvegicus/geneview?gene=ENSRNOG00000039902) | limb-bud and heart [Source:RefSeq_peptide;Acc:NP_084275] | MGI:1925139 | [ENSMUSG00000024063](http://www.ensembl.org/Mus_musculus/geneview?gene=ENSMUSG00000024063) | MGI:1925139 |

Of the eighteen, only the transcription factor E2F6 was represented by a binding site motif in the Transfac database v9.4 (<http://www.gene-regulation.com/pub/databases.html>).

Upstream 5 KB sequences for the 8 genes that had eQTLs in this hotspot were examined for hits to any of the E2F motifs in Transfac using the Transfac "match" software with options for minimizing the false positive rate using high quality motif matrices. Sequences were masked with rat-specific repeats using the default options of the RepeatMasker software (<http://www.repeatmasker.org/>).

Two of the eight genes had hits to the E2F motif:

1. GE18792 ENSRNOG00000006777

MBOA2 Membrane-bound O-acyltransferase domain-containing protein 2

2. GE19375 ENSRNOG00000012757

NEK5 Serine/threonine-protein kinase Nek5 (NimA-related protein kinase 5)

It should be noted, however, that E2F sites are frequent within the genome.

| Table 2. Distribution of the covariates that were used for the multivariate model. The last two columns show the percent of subjects within each population that have the specific trait. | | |
| --- | --- | --- |
| **Covariate** | **Montreal** | **Sydney** |
|
| Number of Caucasian subjects | 545 | 242 |
| Age | 37.6(0.5) | 38.6(0.9) |
| Gender (% Male) | 51% | 100% |
| Non-smoker | 22% | 43% |
| Past smoker | 18% | 24% |
| Current smoker | 60% | 33% |
| Lifetime history of alcohol dependence | 57% | 52% |
| Lifetime history of alcohol abuse | 49% | 50% |
| Alcohol dependence in past year | 41% | 39% |
| Alcohol abuse in past year | 40% | 41% |
| Family history of alcohol dependence in 1st degree relative | 47% | 23% |
| Familial dependence | 34% | 14% |
| Lifetime history of major depression | 29% | 9% |
| Family history of depression in 1st degree relative | 27% | 9% |
| Familial depression | 13% | 2% |
| Familial depression and history of alcohol dependence | 11% | 2% |
| Familial dependence and depression | 13% | 2% |
| Used any antidepressant in last month | 8% | 1% |
| Used medication other than antidepressant in last month | 68% | 33% |

| Table 3. Demographics of Human Subjects | | |
| --- | --- | --- |
|  | **Montreal** | **Sydney** |
| Subjects | 605 | 285 |
| Sex |  |  |
| Males | 313 | 285 |
| Females | 292 | 0 |
| Ethnicity |  |  |
| White | 545 | 242 |
| Black | 12 | 5 |
| Asian | 25 | 32 |
| Other | 23 | 5 |
| Age |  |  |
| <35 | 278 | 130 |
| 35-55 | 279 | 118 |
| >55 | 48 | 37 |
| Education |  |  |
| No high school | 10 | 5 |
| Some high school | 31 | 55 |
| High school | 150 | 19 |
| Undergraduate | 355 | 166 |
| Postgraduate | 58 | 39 |
| Living area |  |  |
| Inner city | 303 | 58 |
| Suburbs | 228 | 191 |
| Rural | 73 | 33 |
| Marital status |  |  |
| Married | 183 | 127 |
| Divorced | 144 | 40 |
| Never married | 277 | 117 |

| Table 4. Haplotype Frequencies and Association with Alcohol Consumption. | | | | | | |
| --- | --- | --- | --- | --- | --- | --- |
| Population | Gene | Haplotype | Haplotype Pattern | Frequency | Percent | Association With Alcohol Consumption |
| Montreal | GAD1 | 1 | CTTCGC | 159.6 | 36.3 |  |
| Montreal | GAD1 | 2 | TCTTGG | 135.3 | 30.8 | recessive model (p<0.0001) |
| Montreal | GAD1 | 3 | TTCCAC | 100.7 | 22.9 |  |
| Montreal | GAD1 | 4 | TTTCGC | 17.1 | 3.9 |  |
| Montreal | GAD1 | 5 | CCTCGC | 12.9 | 2.9 |  |
| Montreal | MPDZ | 1 | ATCGGGT | 543.6 | 62.5 |  |
| Montreal | MPDZ | 2 | GCTCCAA | 111.6 | 12.8 | Recessive model (p<0.0001) |
| Montreal | MPDZ | 3 | GCTCCAT | 96.4 | 11.1 |  |
| Montreal | MPDZ | 4 | GTCCCAT | 33.5 | 3.9 |  |
| Montreal | MPDZ | 5 | GTCGGGT | 26.2 | 3.0 |  |
| Montreal | MPDZ | 6 | GCTCCGT | 8.4 | 1.0 |  |
| Montreal | MPDZ | 7 | ATCCCAT | 6.2 | 0.7 |  |
| Montreal | MPDZ | 8 | ACCGGGT | 5.6 | 0.7 |  |
| Montreal | MPDZ | 9 | ATCGGAT | 5.2 | 0.6 |  |
| Montreal | MPDZ | 10 | GCTCCGA | 5.1 | 0.6 |  |
| Sydney | CHRM5 | 1 | AAGC | 242.9 | 67.1 |  |
| Sydney | CHRM5 | 2 | GGAA | 60.6 | 16.7 |  |
| Sydney | CHRM5 | 3 | AGGC | 32.9 | 9.1 |  |
| Sydney | CHRM5 | 4 | AGGA | 20.3 | 5.6 |  |
| Sydney | GABRB2 | 1 | CTTC | 236.5 | 65.3 |  |
| Sydney | GABRB2 | 2 | TTTT | 54.2 | 15.0 |  |
| Sydney | GABRB2 | 3 | TTTC | 35.3 | 9.8 |  |
| Sydney | GABRB2 | 4 | TCCC | 33.9 | 9.4 |  |
| Sydney | MAPK1 | 1 | GCTGTTTGCCTTAC | 174.9 | 48.3 |  |
| Sydney | MAPK1 | 2 | GTCATCCAACCTGT | 60.0 | 16.6 |  |
| Sydney | MAPK1 | 3 | ATCAGCCACCCTGT | 50.5 | 14.0 | Additive/dominant model* (p<0.0001) |
| Sydney | MAPK1 | 4 | GCTATCTGCCCCGT | 22.0 | 6.1 |  |
| Sydney | MAPK1 | 5 | ATCAGCCACTCTGT | 18.0 | 5.0 |  |
| Sydney | MAPK1 | 6 | GTCATCCACCCTGT | 14.7 | 4.1 |  |
| Sydney | MAPK1 | 7 | GCTATCTGCCCTGT | 12.3 | 3.4 |  |
| Sydney | PPP1R1B | 1 | GT | 301.2 | 83.2 | Dominant model (p=0.0007) |
| Sydney | PPP1R1B | 2 | AC | 47.1 | 13.0 |  |
| Sydney | PPP1R1B | 3 | GC | 13.8 | 3.8 |  |
|  |  |  |  |  |  |  |
| Shown are the haplotype frequencies for the known haplotype blocks containing the SNPs that were significantly associated with alcohol consumption. The additive, dominant and recessive models were explored for association with alcohol consumption at the haplotype level. Haplotypes that showed a significant association (p<0.001) are indicated in the last column.  * Both the additive and dominant effects for this haplotype were significant, indicating that a model with separate effects for each of the three possible combinations fit the data best. | | | | | | |

**Figure 1.** Distribution of alcohol consumption among Caucasian Montreal males (A) and Caucasian Sydney Males (B) in grams of alcohol per kilogram of body weight per day. Levels of alcohol consumption for light, moderate, and heavy drinkers were defined by the estimation of g/kg/day for an 80 kg man using the following criteria: <7 drinks per week = light; 7 to 21 drinks per week = moderate; and more than 21 drinks per week = heavy. The first bar represents the portion of subjects that are light drinkers. The second bar represents the portion of subjects that are moderate drinkers. The remaining bars represent heavy drinkers.


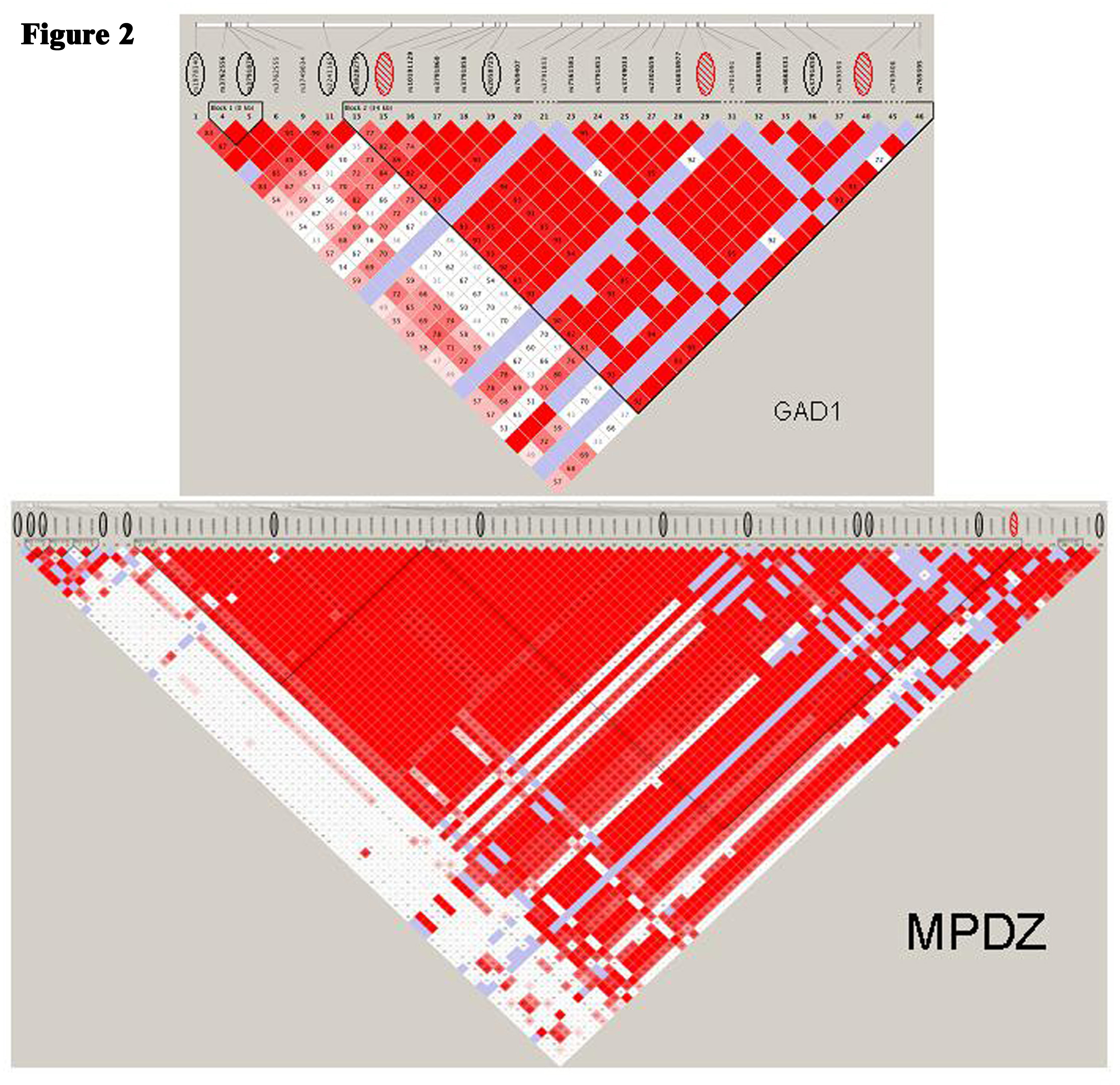


**Figure 2A.**

**Figure 2.** Haplotype block assessment using the CEPH population HapMap data. The circled SNP represent SNPs that are genotyped identifiers in our human data set. The SNP identifiers that are circled in red and with cross-hatching through the circle are SNPs that were significant in: (A) our Montreal population (for genes GAD1 and MPDZ), and (B) our Sydney population (for genes CHRM5, MAPK1 and GABRB2), using a univariate genotype model and an FDR criterion of 0.05.


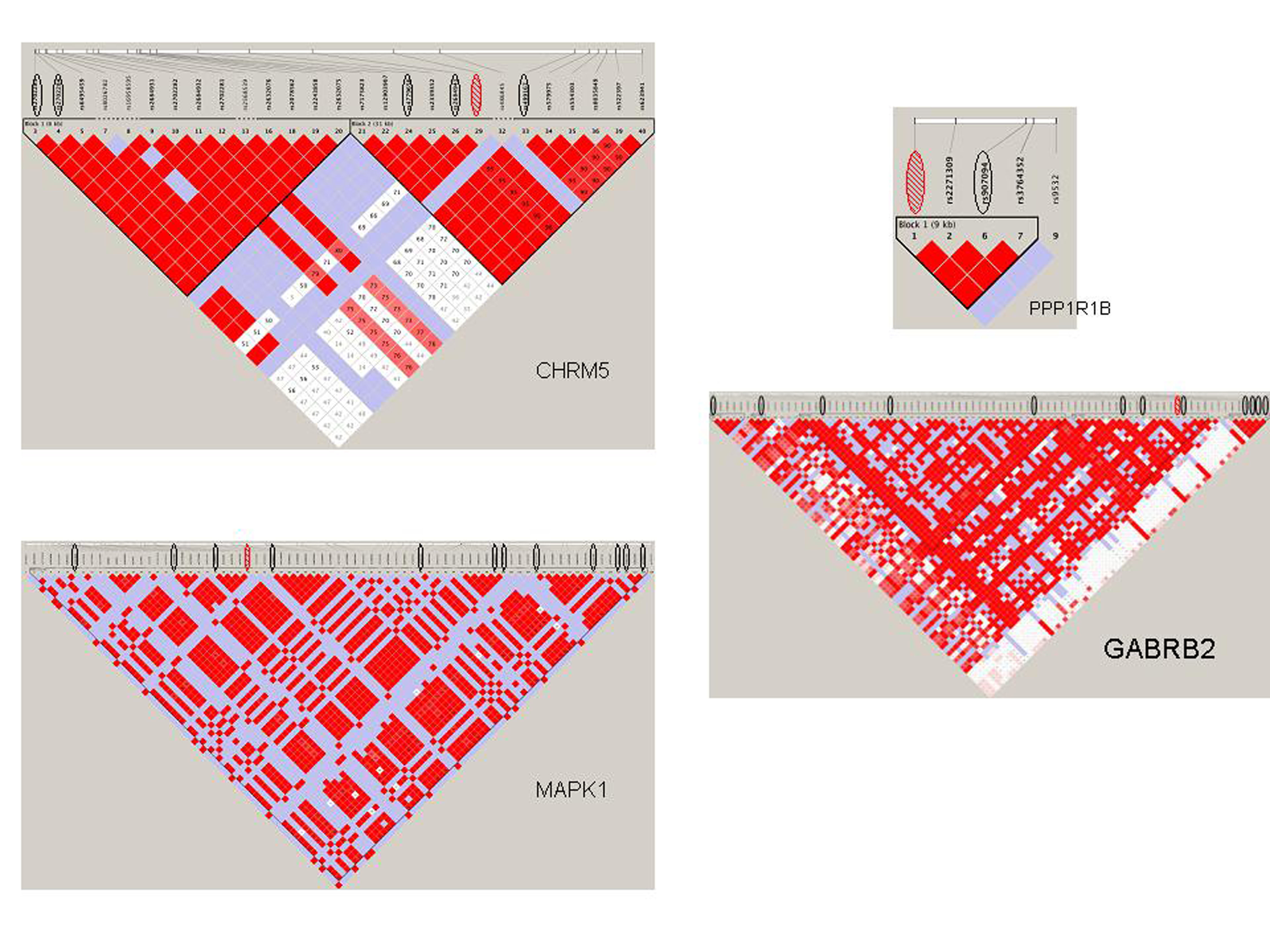


**Figure 2B.**

**Figure 3.** Relationship between alcohol dependence and alcohol consumption. In (A) each bar represents the proportion of Causasian Montreal males who drank within the specified range and who were also diagnosed as having been alcohol dependent within the last 12 months. Similarly, in (B), each bar represents the proportion of Causasian Sydney males who drank within the specified range and who were also diagnosed as having been alcohol dependent within the last 12 months.
